# Coronary Plaque in People With HIV vs Non-HIV Asymptomatic Community and Symptomatic Higher-Risk Populations

**DOI:** 10.1016/j.jacadv.2024.100968

**Published:** 2024-05-03

**Authors:** Julia Karady, Michael T. Lu, Göran Bergström, Thomas Mayrhofer, Jana Taron, Borek Foldyna, Kayla Paradis, Sara McCallum, Judith A. Aberg, Judith S. Currier, Kathleen V. Fitch, Evelynne S. Fulda, Gerald S. Bloomfield, Edgar T. Overton, Lars Lind, Carl Johan Östgren, Olof Elvstam, Stefan Söderberg, Tomas Jernberg, Rosalie Pepe, Michael P. Dubé, David Mushatt, Carl J. Fichtenbaum, Carlos Malvestutto, Markella V. Zanni, Udo Hoffmann, Heather Ribaudo, Steven K. Grinspoon, Pamela S. Douglas

**Affiliations:** a Cardiovascular Imaging Research Center, Massachusetts General Hospital & Harvard Medical School, Boston, Massachusetts, USA; b Cardiovascular Imaging Research Group, Heart and Vascular Center, Semmelweis University, Budapest, Hungary; c Department of Molecular and Clinical Medicine, Institute of Medicine, Sahlgrenska Academy, University of Gothenburg, Gothenburg, Sweden; d Department of Clinical Physiology, Region Västra Götaland, Sahlgrenska University Hospital, Gothenburg, Sweden; e School of Business Studies, Stralsund University of Applied Sciences, Stralsund, Germany; f Faculty of Medicine, Department of Radiology, Medical Center-University of Freiburg, University of Freiburg, Freiburg im Breisgau, Germany; g Metabolism Unit, Massachusetts General Hospital & Harvard Medical School, Boston, Massachusetts, USA; h Department of Medicine, Icahn School of Medicine at Mount Sinai, New York, New York, USA; i Department of Medicine, University of California at Los Angeles, Los Angeles, California, USA; j Duke Clinical Research Institute, Duke University School of Medicine, Durham, North Carolina, USA; k Division of Infectious Diseases, University of Alabama at Birmingham, Birmingham, Alabama, USA; l Division of Clinical Epidemiology, Department of Medical Sciences, Uppsala University, Uppsala, Sweden; m Faculty of Medicine and Health Sciences, Department of Health, Medicine and Caring Sciences, Linköping University, Linköping, Sweden; n Center of Medical Image Science and Visualization, Linköping University, Linköping, Sweden; o Department of Translational Medicine, Lund University, Malmö, Sweden; p Department of Infectious Diseases, Växjö Central Hospital, Växjö, Sweden; q Section of Medicine, Department of Public Health and Clinical Medicine, Umeå University, Umeå, Sweden; r Division of Cardiovascular Medicine, Department of Clinical Sciences, Danderyd Hospital, Karolinska Institute, Stockholm, Sweden; s Cooper University Hospital, Camden, New Jersey, USA; t Division of Infectious Diseases, University of Southern California Keck School of Medicine, Los Angeles, California, USA; u Section of Infectious Disease, Tulane School of Medicine, New Orleans, Louisiana, USA; v Division of Infectious Diseases, University of Cincinnati, Cincinnati, Ohio, USA; w Division of Infectious Diseases, The Ohio State University Wexner Medical Center, Columbus, Ohio, USA; x Innovative Imaging Consulting LLC, Boston, Massachusetts, USA; y Center for Biostatistics in AIDS Research, Harvard T.H. Chan School of Public Health, Boston, Massachusetts, USA

**Keywords:** asymptomatic community cohort, cardiovascular disease, coronary CT angiography, coronary plaque, people with HIV, stable chest pain

## Abstract

**Background:**

People with HIV (PWH) have a high burden of coronary plaques; however, the comparison to people without known HIV (PwoH) needs clarification.

**Objectives:**

The purpose of this study was to determine coronary plaque burden/phenotype in PWH vs PwoH.

**Methods:**

Nonstatin using participants from 3 contemporary populations without known coronary plaques with coronary CT were compared: the REPRIEVE (Randomized Trial to Prevent Vascular Events in HIV) studying PWH without cardiovascular symptoms at low-to-moderate risk (n = 755); the SCAPIS (Swedish Cardiopulmonary Bioimage Study) of asymptomatic community PwoH at low-to-intermediate cardiovascular risk (n = 23,558); and the PROMISE (Prospective Multicenter Imaging Study for Evaluation of Chest Pain) of stable chest pain PwoH (n = 2,291). The coronary plaque prevalence on coronary CT was compared, and comparisons were stratified by 10-year atherosclerotic cardiovascular disease (ASCVD) risk, age, and coronary artery calcium (CAC) presence.

**Results:**

Compared to SCAPIS and PROMISE PwoH, REPRIEVE PWH were younger (50.8 ± 5.8 vs 57.3 ± 4.3 and 60.0 ± 8.0 years; *P* < 0.001) and had lower ASCVD risk (5.0% ± 3.2% vs 6.0% ± 5.3% and 13.5% ± 11.0%; *P* < 0.001). More PWH had plaque compared to the asymptomatic cohort (48.5% vs 40.3%; *P* < 0.001). When stratified by ASCVD risk, PWH had more plaque compared to SCAPIS and a similar prevalence of plaque compared to PROMISE. CAC = 0 was more prevalent in PWH (REPRIEVE 65.2%; SCAPIS 61.6%; PROMISE 49.6%); among CAC = 0, plaque was more prevalent in PWH compared to the PwoH cohorts (REPRIEVE 20.8%; SCAPIS 5.4%; PROMISE 12.3%, *P* < 0.001).

**Conclusions:**

Asymptomatic PWH in REPRIEVE had more plaque than asymptomatic PwoH in SCAPIS but had similar prevalence to a higher-risk stable chest pain cohort in PROMISE. In PWH, CAC = 0 does not reliably exclude plaque.

HIV affects around 38 million people worldwide.^1^ People with HIV (PWH) live longer due to the development of successful antiretroviral therapy. However, PWH often demonstrates an accelerated development of chronic diseases, including increased rates of cardiovascular disease.^2^ The pathomechanism of accelerated cardiovascular disease development among PWH is not fully understood but is explained in part by increased systemic inflammation and residual immune activation. Among PWH, cardiovascular disease may occur at a relatively young age, despite the lower traditional cardiovascular risk profile. After controlling for traditional cardiovascular risk factors, the rate of cardiovascular events, such as myocardial infarction or stroke, is nearly doubled in PWH compared to people without known HIV (PwoH).^3^^,^^4^ PWH develop substantial coronary artery disease (CAD) burden and vulnerable plaque features when compared to individuals without HIV at similar cardiovascular risk, partly attributed to the increased systemic inflammatory activity.^5^, ^6^, ^7^ Moreover, among a large primary prevention cohort of individuals with well-controlled HIV, noncalcified, nonobstructive, and vulnerable plaques were common.^8^

In other non-HIV populations, it is well understood that underlying cardiovascular disease burden is strongly associated with concurrent traditional cardiovascular risk factors. Several large, multicenter studies have recently characterized coronary plaque phenotypes by using coronary computed tomography (CT) imaging in populations with and without HIV and without known CAD.^9^^,^^10^ As a measure of overall cardiovascular risk, the 10-year atherosclerotic cardiovascular disease (ASCVD) risk profile was shown to be associated with the amount and severity of coronary artery plaque, as expected.

However, little is known about the prevalence and phenotype of atherosclerotic coronary plaques in PWH when compared to non-HIV populations. This study aims to compare the burden and phenotype of atherosclerotic coronary plaque in a primary prevention cohort of PWH—without cardiovascular disease symptoms at low-to-intermediate cardiovascular risk—to asymptomatic and symptomatic PwoH to better understand differences in coronary plaque characteristics between PWH and PwoH.

## Methods

### Study population

Three contemporary multicenter clinical trial populations with and without a known HIV and without known history of coronary plaque who underwent coronary CT imaging were included. We studied participants recruited in the following clinical trials: first, a primary prevention PWH cohort at low-to-moderate cardiovascular risk enrolled in the mechanistic CT substudy of the REPRIEVE (Randomized Trial to Prevent Vascular Events in HIV) (n = 755);^11^ second, an asymptomatic community cohort at low-intermediate cardiovascular risk from the SCAPIS (Swedish Cardiopulmonary Bioimage Study) (n = 23,558);^9^ and finally, a symptomatic stable chest pain cohort at intermediate risk from the PROMISE (Prospective Multicenter Imaging Study for Evaluation of Chest Pain) (n = 2,291).^10^ We provide the detailed study-specific inclusion and exclusion criteria in Supplemental Table 1 of the 3 trials. Given that current statin use was an exclusion criterion of REPRIEVE, in the primary analysis we included only nonstatin users from the SCAPIS (93.6%, n = 23,558/25,181) and PROMISE (52.0%, n = 2,291/4,403) trials of PwoH. In a sensitivity analysis, we included all SCAPIS and PROMISE participants, irrespective of their statin status.

Studies were approved by local and central review boards, and participants provided written informed consent for the original studies.

### Coronary CT end points

Standard electrocardiogram (ECG)-gated noncontrast CT studies were used in the 3 populations for the measurement of coronary artery calcium (CAC) score according to the Agatston method.^12^ Coronary CT angiography (CTA) images were acquired using either retrospective ECG-gated or prospectively ECG-triggered protocols according to local protocols and guidelines for the assessment of coronary plaque. Coronary CTA images were interpreted by using the 18-segment coronary segment model.^13^ A coronary plaque resulting in ≥50% stenosis severity was defined as an obstructive disease.^14^ For REPRIEVE and PROMISE, coronary CT datasets were analyzed at the same U.S. central core laboratory.^8^^,^^15^ For SCAPIS, CTs were interpreted at the participating sites.^9^

CAC score, the prevalence of any coronary plaque on coronary CTA, and obstructive plaque (≥50% stenosis) were compared as assessed on the study entry coronary CT.

### Statistical analysis

Descriptive statistics are reported as mean ± SD for continuous variables and as absolute and relative frequencies for categorical variables. Comparisons were stratified by estimated 10-year ASCVD^16^ risk, sex, and age. For statistical comparisons, a 2-sample *t* test was used for continuous variables, and a Fisher exact test was used for categorical variables, and a 2-sided *P* value of ≤0.05 was considered statistically significant. Statistical analyses were performed in Stata 16.1 (StataCorp LP, College Station, TX).

## Results

### Population

Baseline characteristics of the populations stratified by sex are summarized in Table 1. PWH in REPRIEVE, when compared to SCAPIS asymptomatic and PROMISE stable chest pain populations, had fewer women (16.4% vs 48.7% and 53.2%, respectively, *P* < 0.001) and were younger (50.8 ± 5.8 vs 57.3 ± 4.3 and 60.0 ± 8.0 years, *P* < 0.001). The rate of current smokers was the highest in REPRIEVE compared to the other populations (24.0% vs 12.1%, *P* < 0.001 and 20.3%, *P* = 0.031), while the rate of former smokers was similar among the 3 trial populations (31.2% vs 34.4% and 31.9%, both *P* > 0.05). REPRIEVE PWH had lower systolic blood pressure than SCAPIS PwoH and PROMISE PwoH (123 ± 13 vs 125 ± 17 and 131 ± 17; *P* = 0.001). PWH had the lowest low-density lipoprotein cholesterol levels (2.8 ± 0.8 vs 3.5 ± 0.9 and 3.2 ± 0.8 mmol/L; *P* < 0.001), the lowest rate of diabetes (0.4% vs 5.0% and 14.6%; *P* < 0.001), and 10-year ASCVD risk (5.0 ± 3.2 vs 6.0 ± 5.3 and 13.5 ± 11.0, *P* < 0.001) compared to asymptomatic and symptomatic PwoH. Similar results were obtained comparing the 3 cohorts irrespective of statin use (Supplemental Table 2).

**Table 1:** Characteristics of REPRIEVE vs SCAPIS vs PROMISE Participants Without Established Coronary Heart Disease Who Underwent Successful Coronary CT Angiography

	REPRIEVE			SCAPIS			PROMISE		
	Total (N = 755)	Men (n = 631)	Women (n = 124)	Total (N = 23,558)	Men (n = 11,471)	Women (n = 12,087)	Total (N = 2,291)	Men (n = 1,073)	Women (n = 1,218)
Demographics									
Age, y	50.8 ± 5.8	50.8 ± 5.8	50.8 ± 6.2	57.3 ± 4.3	57.2 ± 4.3	57.3 ± 4.3	60.0 ± 8.0	58.2 ± 8.1	61.6 ± 7.7
Women	124 (16.4)	0 (0.0)	124 (100.0)	11,471 (48.7)	0 (0.0)	12,087 (100.0)	1,218 (53.2)	0 (0.0)	1,218 (100.0%)
Body mass index, kg/m^2^	27.3 ± 4.4	26.9 ± 4.1	29.5 ± 5.2	26.7 ± 4.2	27.14 ± 3.77	26.24 ± 4.59	30.2 ± 6.0	30.3 ± 5.4	30.2 ± 6.5
Race									
Black/African American	267 (35.4)	197 (31.2)	70 (56.5)	N/A^a^	N/A^a^	N/A^a^	269 (11.8)	114 (10.7)	155 (12.8)
Asian	10 (1.3)	8 (1.3)	2 (1.6)	N/A^a^	N/A^a^	N/A^a^	66 (2.9)	35 (3.3)	31 (2.6)
White	406 (53.8)	365 (57.8)	41 (33.1)	N/A^a^	N/A^a^	N/A^a^	1,889 (83.1)	898 (84.2)	991 (82.1)
Other^b^	72 (9.5)	61 (9.7)	11 (8.9)	N/A^a^	N/A^a^	N/A^a^	49 (2.2)	19 (1.8)	30 (2.5)
Ethnicity									
Hispanic	182 (24.1)	149 (23.6)	33 (26.6)	N/A^a^	N/A^a^	N/A^a^	150 (6.5)	75 (7.0)	75 (6.2)
Non-Hispanic	563 (74.6)	472 (74.8)	91 (73.4)	N/A^a^	N/A^a^	N/A^a^	2,128 (92.9)	991 (92.4)	1,137 (93.3)
Unknown	10 (1.3)	10 (1.6)	0 (0.0)	N/A^a^	N/A^a^	N/A^a^	13 (0.6)	7 (0.7)	6 (0.5)
Smoking status									
Current smoker	181 (24.0)	150 (23.8)	31 (25.2)	2,857 (12.1)	1,375 (12.0)	1,482 (12.3)	464 (20.3)	259 (24.1)	205 (16.8)
Former smoker	235 (31.2)	198 (31.4)	37 (30.1)	8,115 (34.4)	3,607 (31.4)	4,508 (37.3)	731 (31.9)	368 (34.3)	363 (29.8)
Treatment									
Antihypertensive medication	149 (19.7)	119 (18.9)	30 (24.2)	3,409 (14.5)	1,709 (14.9)	1,700 (14.1)	858 (37.5)	421 (39.2)	437 (35.9)
Blood pressure, mm Hg									
Systolic	123 ± 13	122 ± 13	123 ± 14	125 ± 17	128 ± 15	123 ± 18	131 ± 17	132 ± 16	130 ± 17
Diastolic	78 ± 9	78 ± 9	77 ± 9	77 ± 10	78 ± 10	77 ± 11	79 ± 10	81 ± 10	78 ± 10
Blood lipids, mmol/L									
Total cholesterol	4.8 ± 0.9	4.8 ± 0.9	4.9 ± 1.0	5.6 ± 1.0	5.4 ± 1.0	5.7 ± 1.0	5.3 ± 1.1	5.1 ± 1.0	5.4 ± 1.1
HDL cholesterol	1.3 ± 0.5	1.3 ± 0.5	1.6 ± 0.5	1.6 ± 0.5	1.4 ± 0.4	1.9 ± 0.5	1.3 ± 0.4	1.2 ± 0.3	1.5 ± 0.4
LDL cholesterol	2.8 ± 0.8	2.8 ± 0.8	2.8 ± 0.8	3.5 ± 0.9	3.5 ± 0.9	3.5 ± 0.9	3.2 ± 0.8	3.1 ± 0.8	3.2 ± 0.9
Triglycerides	1.5 ± 1.0	1.6 ± 1.0	1.3 ± 0.6	1.2 ± 0.8	1.3 ± 0.9	1.1 ± 0.0.6	1.7 ± 2.0	1.9 ± 2.6	1.5 ± 1.1
Diabetes	3 (0.4)	3 (0.5)	0 (0.0)	1,178 (5.0)	746 (6.5)	432 (3.6)	335 (14.6)	153 (14.3)	182 (14.9)
Estimated 10-y atherosclerotic cardiovascular risk, %									
Pooled cohort equation	5.0 ± 3.2	5.5 ± 3.1	2.7 ± 2.1	6.0 ± 5.3	8.9 ± 5.7	3.2 ± 2.7	13.5 ± 11.0	16.1 ± 10.8	11.2 ± 10.6

Values are mean ± SD or n (%).

CT = computed tomography; HDL = high-density lipoprotein; LDL = low-density lipoprotein; PROMISE = Prospective Multicenter Imaging Study for Evaluation of Chest Pain; REPRIEVE = Randomized Trial to Prevent Vascular Events in HIV; SCAPIS = Swedish Cardiopulmonary Bioimage Study.

a SCAPIS does not register data on race or ethnic background. However, the majority of the participants were born in Sweden (ca 84%) or other European countries (ca 10%), with ca 2.5% representation from the Middle-East.

b Other race includes participants self-identifying as Native or Indigenous to the enrollment region; more than one race (with no single race noted as predominant); or of an unknown race.

### Presence of CAD

**Figure 1:**
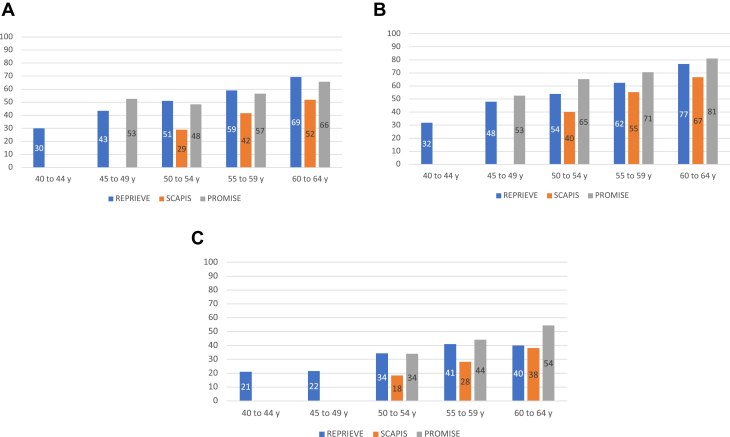
Presence of Any Coronary Atherosclerotic Plaque in REPRIEVE vs SCAPIS vs PROMISE Participants Without Established Coronary Heart Disease as Stratified by Age (A) Total population. (B) Men. (C) Women. PROMISE = Prospective Multicenter Imaging Study for Evaluation of Chest Pain; REPRIEVE = Randomized Trial to Prevent Vascular Events in HIV; SCAPIS = Swedish Cardiopulmonary Bioimage Study.

Any form of coronary plaque was more prevalent in PWH than in the asymptomatic PwoH cohort (48.5% vs 40.3%; *P* < 0.001). When stratified by age groups, coronary plaque was more prevalent in REPRIEVE as compared to PwoH in SCAPIS (*P* < 0.001, across all age groups) (Figure 1A). Further, across age groups, plaque prevalence was similar to the symptomatic population (Figure 1A). Among male participants, compared to PWH stable chest pain, PwoH in PROMISE had more and asymptomatic PwoH in SCAPIS had less prevalent coronary plaque. Among women, PWH in REPRIEVE had similar amounts of prevalent plaque as stable chest pain PwoH in PROMISE but more than asymptomatic PwoH in SCAPIS (Figures 1B and 1C). The prevalence of plaque in REPRIEVE PWH was similar to SCAPIS PwoH participants who were 10 years older; for example, the prevalence of plaque was similar between the 40 and 44-year group in REPRIEVE and the 50 and 54-year group in SCAPIS and between the 50 and 54-year group in REPRIEVE and the 60 and 64-year group in SCAPIS (Figure 1A).

These results were consistent when considering all participants in the PwoH cohorts, including those on statin therapy (Supplemental Figures 1A to 1C).

**Figure 2:**
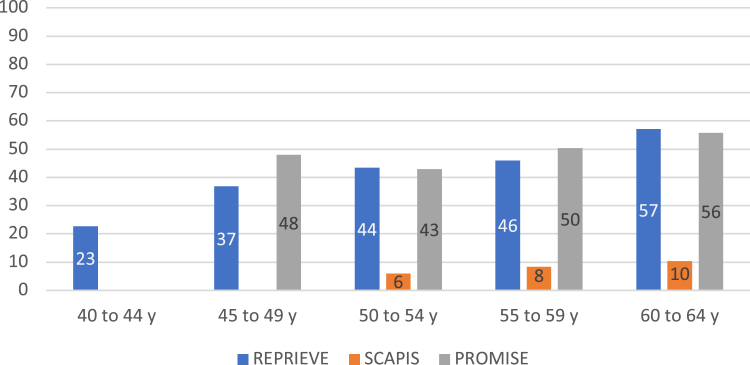
**Presence of Any Noncalcified Coronary Atherosclerotic Plaque of REPRIEVE vs SCAPIS vs PROMISE Participants Without Established Coronary Heart Disease as Stratified by Age** ASCVD = atherosclerotic cardiovascular disease; CAD = coronary artery disease; PROMISE = Prospective Multicenter Imaging Study for Evaluation of Chest Pain; REPRIEVE = Randomized Trial to Prevent Vascular Events in HIV; SCAPIS = Swedish Cardiopulmonary Bioimage Study.

When stratified by age, the presence of any noncalcified plaque was lower in asymptomatic PwoH but similar between PWH and stable chest pain PwoH (Figure 2). Similar results were obtained comparing the 3 cohorts, irrespective of statin use (Supplemental Table 3). Prevalent obstructive plaque was the lowest in REPRIEVE (3.3%) and was more prevalent among PwoH in SCAPIS (4.7%) and almost 4 times higher among PwoH in PROMISE (10.8%) (Supplemental Table 3). Similar trends were observed when considering the total PwoH populations (Supplemental Table 4).

**Figure 3:**
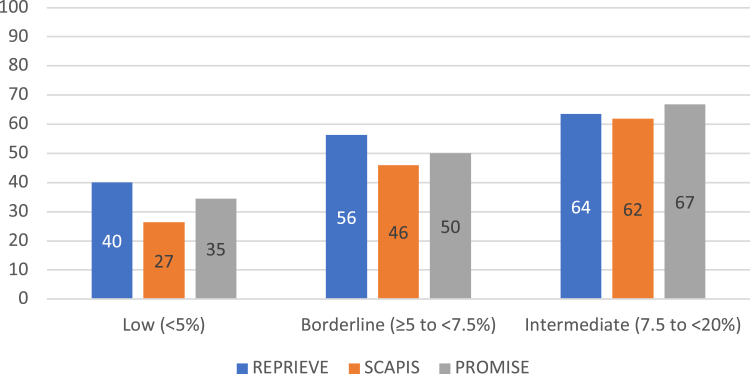
**Presence of Any Coronary Atherosclerotic Plaque of REPRIEVE vs SCAPIS vs PROMISE Participants Without Established Coronary Heart Disease as Stratified by 10-Year Risk for Atherosclerotic Cardiovascular Disease** PROMISE = Prospective Multicenter Imaging Study for Evaluation of Chest Pain; REPRIEVE = Randomized Trial to Prevent Vascular Events in HIV; SCAPIS = Swedish Cardiopulmonary Bioimage Study.

When stratified by low (<5%), borderline (5%-7.5%), and intermediate (7.5%-20%) ASCVD risk, the prevalence of plaque in REPRIEVE PWH in the low (<5%) and borderline risk strata (≥5 to <7.5%) was higher than among asymptomatic PwoH and not statistically significantly higher when compared to symptomatic PwoH (40.1% and 56.3% in REPRIEVE vs 26.5% and 46.0% in SCAPIS, *P* < 0.01, and 34.5% and 50.0% in PROMISE, *P* = 0.09) (Figure 3). In contrast, in the intermediate (≥7.5% to <20%) risk strata, the prevalence of any plaque was similar across the 3 cohorts (*P* > 0.44) (Figure 3).

When considering the full PwoH cohorts independent of statin use, the prevalence of CAD in REPRIEVE was higher than among SCAPIS participants but similar compared to PwoH PROMISE (Supplemental Figure 3).

### CAC = 0 subgroup

**Figure 4:**
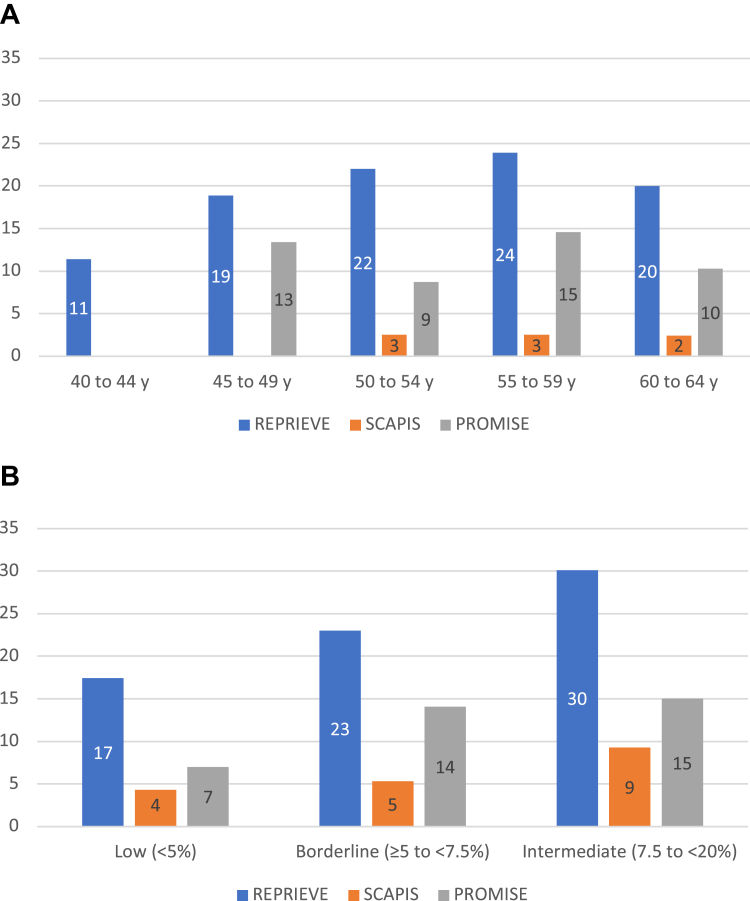
Prevalence of Any Atherosclerotic Plaque in the Subgroup with CAC = 0 (A) Stratified by Age. (B) Stratified by 10-year risk for atherosclerotic cardiovascular disease. ASCVD = atherosclerotic cardiovascular disease; CAC = coronary artery calcium score; CAD = coronary artery disease.

Most of REPRIEVE (65.2%), SCAPIS (61.6%), and nearly half of PROMISE (49.6%) participants had CAC = 0. In this subgroup, plaques were significantly more prevalent in PWH (REPRIEVE 20.8% vs SCAPIS 5.4% [*P* < 0.001] and vs PROMISE 12.3% [*P* < 0.001]). Further, when stratified by age and 10-year ASCVD risk, the prevalence of coronary plaque was significantly greater in PWH compared to low, borderline, and intermediate-risk asymptomatic (*P* < 0.001) and symptomatic (*P* < 0.05) PwoH (Figures 4A and 4B). In our sensitivity analysis, we observed the same results among all SCAPIS and PROMISE participants in contrast to REPRIEVE participants (Supplemental Figure 4).

## Discussion

A large primary prevention cohort of PWH with well-controlled HIV (REPRIEVE) had a greater burden of coronary plaque than a community cohort at similar cardiovascular risk (SCAPIS) and a similar burden of coronary plaque to a stable chest pain population at increased cardiovascular risk (PROMISE). As assessed among patients with CAC = 0, exclusively noncalcified plaque was more common in REPRIEVE than in SCAPIS, and a substantial proportion (21%) of low-risk PWH with CAC = 0 had atherosclerotic plaque, more than in the PwoH cohorts.

These results corroborate and extend prior findings describing premature and prevalent coronary plaque among PWH. In the large case-control study by Guaraldi et al,^17^ an approximately 10-year shift in coronary atherosclerotic risk factor occurrence was observed among PWH compared to age, sex, and gender-matched PwoH. In our analysis—the first comparison assessing anatomically phenotyped coronary plaque occurrence by coronary CT imaging of large PWH vs PwoH cohorts—premature coronary atherosclerotic disease occurred 10 years earlier among PWH than in an asymptomatic community cohort. Further, this is the first time that plaque prevalence among PWH at low cardiovascular risk has been compared to symptomatic individuals at increased cardiovascular risk, and we observed a similar prevalence of plaque across different age groups. Stratifying by ASCVD risk group, we saw a higher prevalence of coronary plaque among PWH at low ASCVD risk compared to symptomatic chest pain participants. This observation corroborates prior findings describing calculators designed for cardiovascular risk estimation to underestimate cardiovascular risk among PWH.^18^ A possible explanation for the latter finding is that in the lower ASCVD risk categories, immune activation and other factors related to HIV infection contribute to the development of atherosclerotic plaques in the absence of traditional risk factors. In this context, the observed similarity in plaque extent between the 3 cohorts in the higher intermediate ASCVD-risk strata, in the presence of increased traditional risk factors, is not surprising.

Prior studies have suggested prevalent coronary plaque among PWH, including the predominance of noncalcified plaques.^5^^,^^6^ In the Multicenter AIDS Cohort Study cohort, HIV-infected men had more prevalent and extensive noncalcified plaque compared to non-HIV males, independent of CAD risk factors.^19^ In this analysis, the presence of any type of noncalcified plaque (including exclusively noncalcified and mixed types of plaques) was similar between PWH and symptomatic PwoH. However, when assessing participants with a CAC = 0, we found a greater prevalence of coronary plaque among PWH when compared to asymptomatic and symptomatic PwoH. This finding may be explained by a greater prevalence of exclusively noncalcified atherosclerotic disease among those with well-controlled HIV compared to those without HIV. In PWH, higher systemic levels of inflammation and immune activation are associated with noncalcified and vulnerable plaque phenotypes.^20^ Because of the high lipid and inflammatory cell content of noncalcified plaques, these plaques are vulnerable and are thus at higher risk of rupture, which may help to explain the higher event rates among PWH. Previously, it has been demonstrated that statin therapy (atorvastatin at 40 mg/day concentration) reduces noncalcified plaque volume and thus could be used for risk modification in PWH; this hypothesis is currently being tested in the REPRIEVE trial.^11^^,^^21^^,^^22^ Also, this finding may imply that CAC, which is a robust tool for cardiovascular risk assessment in the general population, may not perform with sufficient certainty among PWH.

### Strengths and limitations

Importantly, all 3 cohorts were free of known cardiovascular disease, and participants were not using statins in the primary analysis. The 2 PwoH cohorts were selected as they represent the range of ASCVD risk among a primary prevention, middle-aged population. However, this approach also has limitations. First, we compared cohort-level data between one PWH and 2 PwoH studies, as individual-level data from SCAPIS were not available due to data use restrictions. Given these restrictions, we were not able to perform an adjusted analysis, and lifestyle and other differences between the United States and Sweden may not be captured in the ASCVD-risk-based comparisons. Second, a U.S. CT core laboratory interpreted the REPRIEVE and PROMISE CTs; in the Swedish cohort, image data was read at the participating sites, which may introduce slight differences including handling of calcium blooming artifacts. Third, the proportion of women in REPRIEVE (16.4%) was lower compared to PROMISE (50.6%) and SCAPIS (51.6%). Fourth, while reasonably expected to be rare, HIV status was not recorded in the SCAPIS and PROMISE trials. Finally, the studies also differed in racial/ethnic composition. White individuals represented 54% of REPRIEVE and 76% of PROMISE, while race was not collected in SCAPIS; SCAPIS participants were “mainly northern European ancestry”.

## Conclusions

Asymptomatic PWH in REPRIEVE had more prevalent coronary artery plaque compared to an asymptomatic community population in SCAPIS, and similar prevalence to an older, higher-risk stable chest pain cohort in PROMISE. In PWH, CAC = 0 does not reliably exclude coronary plaque due to the higher prevalence of exclusively noncalcified atherosclerotic disease (Central Illustration).

PERSPECTIVES **COMPETENCY IN MEDICAL KNOWLEDGE:** We assessed the question that how coronary plaque prevalence in people with HIV (PWH) without cardiovascular disease symptoms compare to asymptomatic and symptomatic populations without known HIV (PwoH). We found that plaque is more prevalent in PWH at low-intermediate cardiovascular risk than in a low-risk asymptomatic cohort and similar to a higher-risk stable chest pain PwoH. In the subgroup with a coronary artery calcium score (CAC) of 0, plaques are more common in PWH than PwoH.

**TRANSLATIONAL OUTLOOK:** These findings support that clinicians should watch out in low-intermediate risk PWH as coronary plaque is common. Further, CAC = 0 may not reliably exclude prevalent plaque, due to a higher prevalence of non-calcified atherosclerotic disease, thus it is of interest to optimize screening in PWH.

**Central Illustration:**
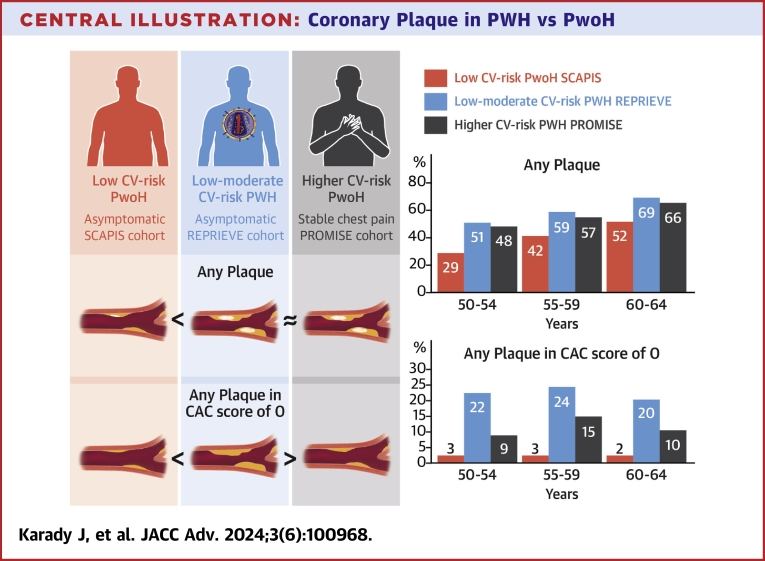
Coronary Plaque in PWH vs PwoH Asymptomatic REPRIEVE PWH at low-to-intermediate cardiovascular risk had more prevalent coronary artery plaque compared to an asymptomatic community PwoH SCAPIS cohort at low cardiovascular risk, and similar prevalence of plaque When compared to an older, higher-risk stable chest pain PwoH PROMISE cohort. PROMISE = Prospective Multicenter Imaging Study for Evaluation of Chest Pain; PWH = people with HIV; PwoH = people without known HIV; REPRIEVE = Randomized Trial to Prevent Vascular Events in HIV; SCAPIS = Swedish Cardiopulmonary Bioimage Study.

## Funding support and author disclosures

10.13039/100000002This paper received NIH grants U01HL123336 to the REPRIEVE Clinical Coordinating Center and U01HL123339 to the REPRIEVE Data Coordinating Center, as well as funding from Kowa Pharmaceuticals, Gilead Sciences, and ViiV Healthcare. The 10.13039/100000060National Institute of Allergy and Infectious Diseases (NIAID) supported this study through grants UM1 AI068636, which supports the AIDS Clinical Trials Group (ACTG) Leadership and Operations Center, and UM1 AI106701, which supports the ACTG Laboratory Center. The PROMISE trial was supported by the 10.13039/100000050National Heart, Lung, and Blood Institute (R01HL098237, R01HL098236, R01HL98305, and R01HL098235). The SCAPIS trial received funding from the Swedish Heart-Lung Foundation, Knut and Alice Wallenberg Foundation, Swedish Research Council and Vinnova (Sweden’s Innovation Agency), University of Gothenburg and Sahlgrenska University Hospital, Karolinska Institutet and Stockholm County Council, Linköping University and University Hospital, Lund University and Skåne University Hospital, Umeå University and University Hospital, and Uppsala University and University Hospital. The content of this manuscript is solely the responsibility of the authors and does not necessarily reflect the views of any of the funding agencies. Dr Lu has received funding to his institution from Kowa, AstraZeneca/MedImmune, Johnson & Johnson Innovation, Ionis, and the American Heart Association unrelated to this research. Dr Taron has received funding by Deutsche Forschungsgesellschaft (DFG, German Research Foundation) (TA 1438/1-2); is on Speakers Bureau for Siemens Healthcare GmbH and Bayer AG; and has received consulting fees from Universimed Cross Media Content GmbH and Core Lab Black Forrest GmbH, unrelated to this work. Dr Foldyna has received funding to his institution from AstraZeneca/MedImmune, MedTrace, and Eli Lilly unrelated to this research. Dr Currier served as an advisor to Merck. Dr Elvstam has received grants to his institution from Pfizer; and has received honoraria as a speaker from Gilead Sciences, unrelated to this research. Dr Dubé has received funding to his institution from Gilead Sciences unrelated to this research. Dr Fichtenbaum has received funding to the institution from ViiV Healthcare, Gilead Sciences, Merck, Cytodyn, and Moderna unrelated to this work and serves on the advisory board for ViiV Healthcare. Dr Malvestutto has received funding to his institution from Lilly; and has received consulting fees from Viiv Healthcare and Gilead Sciences unrelated to this work. Dr Zanni reports being PI on research grants from the NIH (NHLBI and NIAID) and Gilead Sciences to her institution. Dr Ribaudo has received grants from Kowa Pharmaceuticals during the conduct of the study, as well as grants from NIH/NIAID, NIH/NHLBI, NIH/NIDDK, and NIH/NIA, outside the submitted work. Dr Grinspoon reports being a part of the Scientific Advisory Board for Marathon Asset Management and consultant Theratechnologies is unrelated to this report; research funds come from Gilead, Viiv, and Kowa through his institution. All other authors have reported that they have no relationships relevant to the contents of this paper to disclose.
