# Supplementary material for: Coronary Plaque in People With HIV vs Non-HIV Asymptomatic Community and Symptomatic Higher-Risk Populations
Source: JACC Adv. 2024 May 3;3(6):100968. doi: 10.1016/j.jacadv.2024.100968 (PMC11198107; doi:10.1016/j.jacadv.2024.100968)
Supplement: Supplemental Tables 1-5 and Figures 1-3 [file mmc1.docx]

**ONLINE SUPPLEMENT**

**SUPPLEMENTAL TABLES**

**Supplemental table 1. *Study Specific Inclusion and Exclusion Criteria.***

| **REPRIEVE and REPRIEVE Mechanistic CT Substudy^1,2^** | |
| --- | --- |
| **Inclusion criteria** | *Main study criteria:*   - Men and women age ≥40 and ≤75 y of age - Documentation of HIV-1 infection - Combination ART for at least 180 d prior to study entry - CD4+ cell count >100 cells/mm^3^ - Fasting LDL-C as follows:   - If ASCVD risk score <7.5%, LDL-C must be b190 mg/dL   - If ASCVD risk score ≥7.5% and ≤10%, LDL-C must be b160 mg/dL   - If ASCVD risk score ≥10% and ≤15%, LDL-C must be b130 mg/dL - Fasting triglycerides <500 mg/dL - Hemoglobin ≥8 g/dL for female participants and ≥9 g/dL for male participants - GFR ≥60 mL/min/1.73 m 2 or CrCl ≥60 mL/min - ALT ≤2.5× ULN - For persons with known chronic active HBV or HCV, calculated FIB-4 score must be ≤3.25 - Female participants of reproductive potential must have a negative serum or urine pregnancy test result - For women of reproductive potential, willingness to use contraceptives as described in the product information for pitavastatin - Ability and willingness of participant or legal representative to provide written informed consent   *Mechanistic CT substudy criteria:*   - Willingness to complete procedures required for the study - GFR ≥60 mL/min/1.73m 2 or CrCl ≥60 mL/min, as per REPRIEVE within 14 days prior to CCTA |
| **Exclusion criteria** | *Main study criteria:*   - Clinical ASCVD, as defined by 2013 ACC/AHA guidelines - Current diabetes mellitus if LDL-C ≥70 mg/dL - 10-y ASCVD risk score estimated by Pooled Cohort Equations >15% - Active cancer within 12 m prior to study entry - Known decompensated cirrhosis - History of myositis or myopathy with active disease in the 180 d prior to study entry - Known untreated symptomatic thyroid disease - History of allergy or severe adverse reaction to statins - Use of specific immunosuppressants or immunomodulatory agents in the 30 d prior to study entry - Current use of erythromycin, colchicine, or rifampin - Use of any statin drugs, gemfibrozil, or PCSK9 inhibitors in the 90 d prior to study entry - Current use of an investigational new drug that would be contraindicated - Serious illness or trauma requiring systemic treatment or hospitalization in the 30 d prior to study entry - Known active or recent (not fully resolved within 30 d prior to study entry) systemic bacterial, fungal, parasitic, - or viral infections (except HIV, HBV, HPV, or HCV) - Current breastfeeding - Alcohol or drug use that, in the opinion of the site investigator, would interfere with completion of study procedures - Other medical, psychiatric, or psychological condition that, in the opinion of the site investigator, would interfere with completion of study procedures and or adherence to study drug   *Mechanistic CT substudy criteria:*   - Known allergy to iodinated contrast agent - Currently symptomatic asthma - Allergy to beta blockers - Contraindication to beta blockers (i.e., taking daily asthma medications) - Positive pregnancy test within 24 hours prior to study entry - Any condition that prohibits the individual from completing the CCTA - BMI ≥40 kg/m 2 - Cardiac arrhythmia at enrollment precluding CCTA; such as atrial fibrillation with heart rate N80 beats per minute or frequent ectopic beats |
| **SCAPIS** | |
| **Inclusion criteria** | - Individuals between 50-65 years of age |
| **Exclusion criteria** | - Individuals who did not undergo CCTA or if there was a technical failure in reading any of the 4 proximal segments (proximal right coronary, left main, proximal left anterior descending [LAD], and proximal circumflex artery) on the CCTA images - Previously had a MI, percutaneous coronary intervention, or coronary artery bypass grafting. |
| **PROMISE^3^** | |
| **Inclusion criteria** | - New or worsening chest pain syndrome or equivalent symptoms suspicious for clinically significant CAD - No prior cardiac evaluation for this episode of symptoms - Planned noninvasive testing for diagnosis - Men age ≥55 y and women age ≥65 y - If age in men 45 to 54 y or women 50 to 64 y, then must have increased probability of CAD due to ≥1 of the following risk factors: - Known significant congenital, valvular (greater than or equal to moderate) or cardiomyopathic process (hypertrophic cardiomyopathy or reduced systolic left ventricular [LV] function [LV ejection fraction <40%]) that could explain cardiac symptoms   - Diabetes mellitus requiring medical treatment   - Peripheral arterial disease, defined as documented peripheral arterial stenosis ≥50%, treated medically or invasively   - Cerebrovascular disease (stroke), defined as documented carotid stenosis ≥50%, treated medically or invasively   - Ongoing tobacco use   - Hypertension   - Abnormal ankle-brachial index, defined as b0.9   - Dyslipidemia   - Body mass index <40 kg/m 2 - Serum creatinine ≤1.5 mg/dL within the past 90 d - Negative urine/serum pregnancy test result for female subjects of childbearing potential |
| **Exclusion criteria** | - Diagnosed or suspected ACS requiring hospitalization or urgent or emergent testing; elevated troponin or CK-MB; outpatients who have completed a rule-out ACS protocol are eligible provided they have 2 sets of negative biomarkers and a nondiagnostic or normal ECG - Hemodynamically or clinically unstable condition (systolic blood pressure [BP] b90 mm Hg, severe atrial or ventricular arrhythmias, or persistent resting chest pain felt to be ischemic despite adequate therapy) - Known CAD with clinical history of MI, percutaneous coronary intervention, coronary artery bypass graft, or any angiographic evidence of CAD ≥50% lesion in a major epicardial vessel - Any invasive coronary angiography or noninvasive anatomical or functional cardiovascular test for detection of CAD, including CCTA and ExECG, within the previous 12 mo (±30 d); prior resting ECG and/or resting echo do not constitute an exclusion to participation - Contraindication to a CCTA, including, but not limited to:   - Allergy to iodinated contrast agent   - Pregnancy - Any other contraindications that would preclude performing a CCTA per local site practice, such as ≥1 of the following:   - Inability to receive β-blockers if heart rate is N65 beats/min   - Agatston score <800   - Cardiac arrhythmia - Life expectancy <2 y - Unable to provide written informed consent or participate in long-term follow-up |

ALT=Alanine Transaminase; ASCVD=Atherosclerotic Cardiovascular Disease; CAD=Coronary Artery Disease; CCTA=Coronary Computed Tomography Angiography; ECG=Electrocardiogram; GFR=Glomerular Filtration Rate; LDL=Low density lipoprotein; MI=Myocardial Infraction

**Supplemental table 2.** Characteristics of REPRIEVE vs SCAPIS vs PROMISE participants without established coronary heart disease who underwent successful coronary CT angiography.

|  | **REPRIEVE** | | | **SCAPIS** | | | **PROMISE** | | |
| --- | --- | --- | --- | --- | --- | --- | --- | --- | --- |
|  | **Total** | **Men** | **Women** | **Total** | **Men** | **Women** | **Total** | **Men** | **Women** |
| Sample size, n | 755 | 631 | 124 | 25182 | 12444 | 12738 | 4403 | 2129 | 2274 |
| **Demographics** | | | | | | | | | |
| Women, n (%) | 124 (16.4) | 0 (0.0) | 124 (100.0) | 12738 (50.6) | 0 (0.0) | 12738 (100.0) | 2274 (51.6) | 0 (0.0) | 2274 (100.0%) |
| Age, y | 50.8±5.8 | 50.8±5.8 | 50.8±6.2 | 57.4±4.3 | 57.4±4.4 | 57.4±4.3 | 60.5±8.2 | 58.5±8.2 | 62.3±7.7 |
| Race, n (%) |  |  |  |  |  |  |  |  |  |
| Black/African American | 267 (35.4) | 197 (31.2) | 70 (56.5) | N/A* | N/A* | N/A* | 455 (10.4) | 188 (8.9) | 267 (11.9) |
| Asian | 10 (1.3) | 8 (1.3) | 2 (1.6) | N/A* | N/A* | N/A* | 132 (3.0) | 71 (3.4) | 61 (2.7) |
| White | 406 (53.8) | 365 (57.8) | 41 (33.1) | N/A* | N/A* | N/A* | 3677 (84.3) | 1805 (85.6) | 1872 (83.1) |
| Other** | 72 (9.5) | 61 (9.7) | 11 (8.9) | N/A* | N/A* | N/A* | 99 (2.3) | 45 (2.1) | 54 (2.4) |
| Ethnicity |  |  |  |  |  |  |  |  |  |
| Hispanic | 182 (24.1) | 149 (23.6) | 33 (26.6) | N/A* | N/A* | N/A* | 333 (7.6) | 167 (7.8) | 166 (7.3) |
| Non-Hispanic | 563 (74.6) | 472 (74.8) | 91 (73.4) | N/A* | N/A* | N/A* | 4048 (91.9) | 1948 (91.5) | 2100 (92.4) |
| Unknown | 10 (1.3) | 10 (1.6) | 0 (0.0) | N/A* | N/A* | N/A* | 22 (0.5) | 14 (0.7) | 8 (0.4) |
| **Anthropometry** | | | | | | | | | |
| Body mass index, kg/m2 | 27.3±4.4 | 26.9±4.1 | 29.5±5.2 | 26.8±4.3 | 27.3±3.8 | 26.3±4.6 | 30.4±5.9 | 30.4±5.4 | 30.4±6.4 |
| **Smoking status, n (%)** | | | | | | | | | |
| Current smoker | 181 (24.0) | 150 (23.8) | 31 (25.2) | 3079 (12.5) | 1495 (12.3) | 1584 (12.6) | 788 (17.9) | 449 (21.1) | 339 (14.9) |
| Former smoker | 235 (31.2) | 198 (31.4) | 37 (30.1) | 8791 (35.6) | 3997 (32.9) | 4794 (38.3) | 1464 (33.3) | 754 (35.4) | 710 (31.2) |
| **Treatment, n (%)** | | | | | | |  |  |  |
| Antihypertensive medication | 149 (19.7) | 119 (18.9) | 30 (24.2) | 4437 (17.6) | 2352 (18.9) | 2085 (16.4) | 1805 (42.9) | 906 (45.5) | 899 (40.5) |
| **Blood pressure, mm Hg** | | | | | | | | | |
| Systolic | 123±13 | 122±13 | 123±14 | 126±17 | 129±15 | 123±18 | 131±17 | 131±16 | 131±17 |
| Diastolic | 78±9 | 78±9 | 77±9 | 78±10 | 78±10 | 77±11 | 79±10 | 80±10 | 77±10 |
| **Blood lipids, mmol/L** | | | | | | | | | |
| Total cholesterol | 4.8±0.9 | 4.8±0.9 | 4.9±1.0 | 5.5±1.0 | 5.4±1.0 | 5.7±1.0 | 5.1±1.2 | 4.9±1.2 | 5.3±1.2 |
| HDL cholesterol | 1.3±0.5 | 1.3±0.5 | 1.6±0.5 | 1.6±0.5 | 1.4±0.4 | 1.9±0.5 | 1.3±0.4 | 1.2±0.3 | 1.5±0.4 |
| LDL cholesterol | 2.8±0.8 | 2.8±0.8 | 2.8±0.8 | 3.5±1.0 | 3.5±0.9 | 3.5±1.0 | 3.0±1.0 | 3.0±0.9 | 3.0±1.0 |
| Triglycerides | 1.5±1.0 | 1.6±1.0 | 1.3±0.6 | 1.2±0.8 | 1.4±0.9 | 1.1±0.6 | 1.7±1.7 | 1.9±2.2 | 1.6±1.2 |
| **10-year Risk of Atherosclerotic Cardiovascular Disease, %** | | | | | | | | | |
| Pooled cohort equation^16^ | 5.0±3.2 | 5.5±3.1 | 2.7±2.1 | 6.2±5.4 | 9.1±5.9 | 3.3±2.9 | 14.3±11.4 | 16.5±11.4 | 12.2±11.1 |

HDL=High density lipoprotein; LDL=Low density lipoprotein.

*SCAPIS does not register data on race or ethnic background. However, the majority of the participants were born in Sweden (ca 84%) or other European countries (ca 10%), with ca 2,5% representation from the Middle-East.

**Other race includes participants self-identifying as Native or Indigenous to the enrollment region; more than one race (with no single race noted as predominant); or of unknown race.

**Supplemental table 3.** Prevalence of CT angiography–detected atherosclerosis and CAC in REPRIEVE vs SCAPIS vs PROMISE participants without established coronary heart disease who underwent successful coronary CT angiography and CAC scoring, stratified by sex and age.

|  |  | **REPRIEVE** | | | | | | | | | | **SCAPIS** | | | | | | | **PROMISE** | | | | | | | |
| --- | --- | --- | --- | --- | --- | --- | --- | --- | --- | --- | --- | --- | --- | --- | --- | --- | --- | --- | --- | --- | --- | --- | --- | --- | --- | --- |
|  | **Total** | **Men** | | | | | **Women** | | | | | **Total** | **Men** | | | **Women** | | | **Total** | **Men** | | | | **Women** | | |
| **Characteristics** |  | **40 to 44 y** | **45 to 49 y** | **50 to 54 y** | **55 to 59 y** | **60 to 64 y** | **40 to 44 y** | **45 to 49 y** | **50 to 54 y** | **55 to 59 y** | **60 to 64 y** |  | **50 to 54 y** | **55 to 59 y** | **60 to 64 y** | **50 to 54 y** | **55 to 59 y** | **60 to 64 y** |  | **45 to 49 y** | **50 to 54 y** | **55 to 59 y** | **60 to 64 y** | **50 to 54 y** | **55 to 59 y** | **60 to 64 y** |
| Sample size | 747 | 91 | 175 | 202 | 117 | 39 | 19 | 37 | 35 | 22 | 10 | 23,558 | 4,088 | 3,817 | 3,566 | 4,258 | 3,964 | 3,865 | 1689 | 173 | 241 | 264 | 180 | 282 | 299 | 250 |
| **CTA** | | | | | | | | | | | | | | | | | | | | | | | | | | |
| Any form of atherosclerosis | 362 (48.5) | 29 (31.9) | 84 (48.0) | 109 (54.0) | 73 (62.4) | 30 (76.9) | 4 (21.0) | 8 (21.6) | 12 (34.3) | 9 (40.9) | 4 (40.0) | 9,503 (40.3) | 1,640 (40.1) | 2,112 (55.3) | 2,378 (66.7) | 779 (18.3) | 1,118 (28.2) | 1,476 (38.2) | 944 (55.9) | 91 (52.6) | 157 (65.2) | 186 (70.5) | 146 (81.1) | 96 (34.0) | 132 (44.2) | 136 (54.4) |
| Any stenosis ≥50% | 24/735 (3.3) | 1/91 (1.1) | 7/172 (4.1) | 6/198 (3.0) | 8/114 (7.0) | 2/39 (5.1) | 0/18 (0.0) | 0/36 (0.0) | 0/35 (0.0) | 0/22 (0.0) | 0/10 (0.0) | 1119 (4.7) | 165 (4.0) | 3.7 (8.0) | 411 (11.5) | 47 (1.1) | 72 (1.8) | 117 (3.0) | 182 (10.8) | 16 (9.3) | 30 (12.5) | 44 (16.7) | 38 (21.1) | 10 (3.6) | 22 (7.4) | 22 (8.8) |
| Any plaque noncalcified | 298 (39.9) | 22 (24.2) | 72 (41.1) | 94 (46.5) | 57 (48.7) | 25 (64.1) | 3 (15.8) | 6 (16.2) | 9 (25.7) | 7 (31.8) | 3 (30.0) | 1915 (8.1) | 333 (8.1) | 453 (11.9) | 504 (14.1) | 156 (3.7) | 199 (5.0) | 270 (7.0) | 832 (49.3) | 83 (48.0) | 141 (58.5) | 170 (64.4) | 130 (72.2) | 84 (29.8) | 114 (38.1) | 110 (44.0) |
| Any noncalcified stenosis ≥50% | 4/735 (0.5) | 0 (0.0) | 1 (0.6) | 1 (0.5) | 2 (1.8) | 0 (0.0) | 0 (0.0) | 0 (0.0) | 0 (0.0) | 0 (0.0) | 0 (0.0) | 285 (1.2) | 42 (1.0) | 75 (2.0) | 90 (2.5) | 15 (0.4) | 23 (0.6) | 40 (1.0) | 43 (2.6) | 4 (2.3) | 7 (2.9) | 12 (4.6) | 7 (3.9) | 2 (0.7) | 6 (2.0) | 5 (2.0) |
| **CAC** | | | | | | | | | | | | | | | | | | | | | | | | | | |
| Sample size | 710 | 82 | 168 | 195 | 107 | 38 | 17 | 37 | 34 | 22 | 10 | 2,3403 | 4,055 | 3,790 | 3,540 | 4,231 | 3,994 | 3,843 | 1475 | 149 | 222 | 230 | 158 | 247 | 250 | 219 |
| 0 | 463 (65.2) | 65 (79.3) | 111 (66.1) | 124 (63.6) | 56 (52.3) | 14 (36.8) | 14 (82.4) | 32 (86.5) | 26 (76.5) | 15 (68.2) | 6 (60.0) | 14,423 (61.6) | 2,508 (61.8) | 1,760 (46.4) | 1246 (35.2) | 3,537 (83.6) | 2,917 (74.0) | 2,455 (63.9) | 732 (49.6) | 82 (55.0) | 91 (41.0) | 90 (39.1) | 33 (20.9) | 173 (70.0) | 150 (60.0) | 113 (51.6) |
| >0 | 247 (34.8) | 17 (20.7) | 57 (33.9) | 71 (36.4) | 51 (47.7) | 24 (63.2) | 3 (17.7) | 5 (13.5) | 8 (23.5) | 7 (31.8) | 4 (40.0) | 8,980 (38.4) | 1,547 (38.2) | 2,030 (53.6) | 2294 (64.8) | 694 (16.4) | 1,027 (26.0) | 1,388 (36.1) | 743 (50.4) | 67 (45.0) | 131 (59.0) | 140 (60.9) | 125 (79.1) | 74 (30.0) | 100 (40.0) | 106 (48.4) |
| 1 to 10 (ultralow) | 67 (9.4) | 5 (6.1) | 20 (11.9) | 22 (11.3) | 8 (7.5) | 4 (10.5) | 1 (5.9) | 2 (5.4) | 2 (5.9) | 3 (13.6) | 0 (0.0) | 2,666 (11.4) | 559 (13.8) | 564 (14.9) | 459 (13.0) | 303 (7.2) | 357 (9.1) | 424 (11.0) | 118 (8.0) | 14 (9.4) | 19 (8.6) | 18 (7.8) | 14 (8.9) | 18 (7.3) | 18 (7.2) | 17 (7.8) |
| 11 to 100 (low) | 109 (15.4) | 9 (11.0) | 26 (15.5) | 32 (16.4) | 22 (20.6) | 10 (26.3) | 2 (11.8) | 2 (5.4) | 2 (5.9) | 1 (4.6) | 3 (30.0) | 3,888 (16.6) | 652 (16.1) | 902 (23.8) | 901 (25.5) | 303 (7.2) | 489 (12.4) | 641 (16.7) | 313 (21.2) | 31 (20.8) | 54 (24.3) | 59 (25.7) | 33 (20.9) | 39 (15.8) | 48 (19.2) | 49 (22.4) |
| 101 to 400 (moderate) | 60 (8.5) | 3 (3.7) | 8 (4.8) | 14 (7.2) | 19 (17.8) | 8 (21.1) | 0 (0.0) | 1 (2.7) | 3 (8.8) | 3 (13.6) | 1 (10.0) | 1747 (7.5) | 262 (6.5) | 410 (10.8) | 603 (17.0) | 71 (1.7) | 150 (3.8) | 251 (6.5) | 193 (13.1) | 16 (10.7) | 36 (16.2) | 36 (15.7) | 36 (22.8) | 16 (6.5) | 26 (10.4) | 27 (12.3) |
| >400 (high) | 11 (1.6) | 0 (0.0) | 3 (1.8) | 3 (1.5) | 2 (1.9) | 2 (5.3) | 0 (0.0) | 0 (0.0) | 1 (2.9) | 0 (0.0) | 0 (0.0) | 679 (2.9) | 74 (1.8) | 154 (4.1) | 331 (9.4) | 17 (0.4) | 31 (0.8) | 72 (1.9) | 119 (8.1) | 6 (4.0) | 22 (9.9) | 27 (11.7) | 42 (26.6) | 1 (0.4) | 8 (3.2) | 13 (5.9) |
| CAC* | 40 (8–112) | 25 (5–69) | 25 (6–68) | 40 (8–100) | 80 (16–214) | 63 (25–126) | 16 (1–68) | 11 (2–18) | 66 (15–329) | 43 (1–146) | 83 (65–117) | 0 (0-14) | 0 (0-9) | 2 (0-39) | 14 (0-112) | 0 (0-0) | 0 (0-1) | 0 (0-11) | 76 (20–224) | 46 (12–163) | 77 (23–214) | 90 (22–247) | 179 (35–602) | 41 (11–95) | 47 (16–135) | 66 (21–189) |

CAC=Coronary Artery Calcium; CTA=Computed Tomography Angiography.

**Supplemental Table 4.** Prevalence of CT angiography–detected atherosclerosis and CAC in REPRIEVE vs SCAPIS vs PROMISE participants without established coronary heart disease who underwent successful coronary CT angiography and CAC scoring, stratified by sex and age.

|  | **REPRIEVE** | | | | | | | | | | | **SCAPIS** | | | | | | | **PROMISE** | | | | | | | |
| --- | --- | --- | --- | --- | --- | --- | --- | --- | --- | --- | --- | --- | --- | --- | --- | --- | --- | --- | --- | --- | --- | --- | --- | --- | --- | --- |
| **Characteristics** | **Total** | **Men** | | | | | **Women** | | | | | **Total** | **Men** | | | **Women** | | | **Total** | **Men** | | | | **Women** | | |
|  |  | **40 to 44 y** | **45 to 49 y** | **50 to 54 y** | **55 to 59 y** | **60 to 64 y** | **40 to 44 y** | **45 to 49 y** | **50 to 54 y** | **55 to 59 y** | **60 to 64 y** |  | **50 to 54 y** | **55 to 59 y** | **60 to 64 y** | **50 to 54 y** | **55 to 59 y** | **60 to 64 y** |  | **45 to 49 y** | **50 to 54 y** | **55 to 59 y** | **60 to 64 y** | **50 to 54 y** | **55 to 59 y** | **60 to 64 y** |
| **CTA** | | | | | | | | | | | | | | | | | | | |  | | | |  | | |
| Sample size | 747 | 91 | 175 | 202 | 117 | 39 | 19 | 37 | 35 | 22 | 10 | 25 182 | 4278 | 4128 | 4038 | 4348 | 4189 | 4201 | 3173 | 348 | 442 | 501 | 379 | 439 | 555 | 509 |
| Any form of atherosclerosis, n (%) | 362 (48.5) | 29 (31.9) | 84 (48.0) | 109 (54.0) | 73 (62.4) | 30 (76.9) | 4 (21.0) | 8 (21.6) | 12 (34.3) | 9 (40.9) | 4 (40.0) | 10 603 (42.1) | 1762 (41.2) | 2337 (56.6) | 2776 (68.7) | 816 (18.8) | 1229 (29.3) | 1683 (40.1) | 1915 (60.4) | 188 (54.0) | 301 (68.1) | 373 (74.5) | 325 (85.8) | 168 (38.3) | 269 (48.5) | 291 (57.2) |
| Any stenosis ≥50%, n (%) | 24/735 (3.3) | 1/91 (1.1) | 7/172 (4.1) | 6/198 (3.0) | 8/114 (7.0) | 2/39 (5.1) | 0/18 (0.0) | 0/36 (0.0) | 0/35 (0.0) | 0/22 (0.0) | 0/10 (0.0) | 1317 (5.2) | 185 (4.3) | 343 (8.3) | 508 (12.6) | 52 (1.2) | 83 (2.0) | 146 (3.5) | 377 (11.9) | 30 (8.6) | 62 (14.0) | 95 (19.0) | 85 (22.4) | 22 (5.0) | 41 (7.4) | 42 (8.3) |
| Any plaque non-calcified, n (%) | 298 (39.9) | 22 (24.2) | 72 (41.1) | 94 (46.5) | 57 (48.7) | 25 (64.1) | 3 (15.8) | 6 (16.2) | 9 (25.7) | 7 (31.8) | 3 (30.0) | 2102 (8.3) | 359 (8.4) | 492 (11.9) | 575 (14.2) | 160 (3.7) | 224 (5.3) | 292 (7.0) | 1681 (53.0) | 166 (47.7) | 274 (62.0) | 327 (65.3) | 289 (76.3) | 150 (34.2) | 230 (41.4) | 245 (48.1) |
| Any non-calcified stenosis ≥50%, n (%) | 4/735 (0.5) | 0 (0.0) | 1 (0.6) | 1 (0.5) | 2 (1.8) | 0 (0.0) | 0 (0.0) | 0 (0.0) | 0 (0.0) | 0 (0.0) | 0 (0.0) | 315 (1.3) | 42 (1.0) | 80 (1.9) | 110 (2.7) | 16 (0.4) | 24 (0.6) | 43 (1.0) | 86 (2.7) | 10 (2.9) | 16 (3.6) | 19 (3.8) | 14 (3.7) | 7 (1.6) | 11 (2.0) | 9 (1.8) |
| **CAC** | | | | | | | | | | | | | | | | | | | |  | | | |  | | |
| Sample size | 710 | 82 | 168 | 195 | 107 | 38 | 17 | 37 | 34 | 22 | 10 | 25 014 | 4243 | 4099 | 4006 | 4320 | 4169 | 4177 | 2764 | 303 | 399 | 427 | 339 | 388 | 471 | 437 |
| 0, n (%) | 463 (65.2) | 65 (79.3) | 111 (66.1) | 124 (63.6) | 56 (52.3) | 14 (36.8) | 14 (82.4) | 32 (86.5) | 26 (76.5) | 15 (68.2) | 6 (60.0) | 14 957 (59.8) | 2583 (60.9) | 1850 (45.1) | 1317 (32.9) | 3592 (83.1) | 3032 (72.7) | 2583 (61.8) | 1242 (44.9) | 160 (52.8) | 148 (37.1) | 144 (33.7) | 54 (15.9) | 258 (66.5) | 263 (55.8) | 215 (49.2) |
| >0, n (%) | 247 (34.8) | 17 (20.7) | 57 (33.9) | 71 (36.4) | 51 (47.7) | 24 (63.2) | 3 (17.7) | 5 (13.5) | 8 (23.5) | 7 (31.8) | 4 (40.0) | 10 057 (40.2) | 1660 (39.1) | 2249 (54.9) | 2689 (67.1) | 728 (16.9) | 1137 (27.3) | 1594 (38.2) | 1522 (55.1) | 143 (47.2) | 251 (62.9) | 283 (66.3) | 285 (84.1) | 130 (33.5) | 208 (44.2) | 222 (50.8) |
| 1 to 10, n (%) | 67 (9.4) | 5 (6.1) | 20 (11.9) | 22 (11.3) | 8 (7.5) | 4 (10.5) | 1 (5.9) | 2 (5.4) | 2 (5.9) | 3 (13.6) | 0 (0.0) | 2867 (11.5) | 582 (13.7) | 610 (14.9) | 500 (12.5) | 313 (7.2) | 393 (9.4) | 469 (11.2) | 207 (7.5) | 25 (8.3) | 36 (9.0) | 26 (6.1) | 22 (6.5) | 28 (66.5) | 38 (8.1) | 32 (7.3) |
| 11 to 100, n (%) | 109 (15.4) | 9 (11.0) | 26 (15.5) | 32 (16.4) | 22 (20.6) | 10 (26.3) | 2 (11.8) | 2 (5.4) | 2 (5.9) | 1 (4.6) | 3 (30.0) | 4287 (17.1) | 704 (16.6) | 979 (23.9) | 1021 (25.5) | 313 (7.2) | 529 (12.7) | 741 (17.7) | 628 (22.7) | 70 (23.1) | 97 (24.3) | 124 (29.0) | 69 (20.4) | 64 (16.5) | 102 (21.7) | 102 (23.3) |
| 101 to 400, n (%) | 60 (8.5) | 3 (3.7) | 8 (4.8) | 14 (7.2) | 19 (17.8) | 8 (21.1) | 0 (0.0) | 1 (2.7) | 3 (8.8) | 3 (13.6) | 1 (10.0) | 2022 (8.1) | 285 (6.7) | 464 (11.3) | 726 (18.1) | 83 (1.9) | 176 (4.2) | 288 (6.9) | 415 (15.0) | 32 (10.6) | 72 (18.1) | 82 (19.2) | 92 (27.1) | 30 (7.7) | 47 (10.0) | 60 (13.7) |
| >400, n (%) | 11 (1.6) | 0 (0.0) | 3 (1.8) | 3 (1.5) | 2 (1.9) | 2 (5.3) | 0 (0.0) | 0 (0.0) | 1 (2.9) | 0 (0.0) | 0 (0.0) | 881 (3.5) | 89 (2.1) | 196 (4.8) | 442 (11.0) | 19 (0.4) | 39 (0.9) | 96 (2.3) | 272 (9.8) | 16 (5.3) | 46 (11.5) | 51 (11.9) | 102 (30.1) | 8 (2.1) | 21 (4.5) | 28 (6.4) |
| CAC, median (IQR) | 40 (8–112) | 25 (5–69) | 25 (6–68) | 40 (8–100) | 80 (16–214) | 63 (25–126) | 16 (1–68) | 11 (2–18) | 66 (15–329) | 43 (1–146) | 83 (65–117) | 35 (8–126) | 25 (5–85) | 36 (9–130) | 75 (18–237) | 17 (4–53) | 21 (6–71) | 30 (8–96) | 82 (23–253) | 46 (15–163) | 85 (25–265) | 95 (26–274) | 210 (60–572) | 45 (13–130) | 50 (15–148) | 69 (24–194) |

CAC=Coronary Artery Calcium; CTA=Computed Tomography Angiography.

Supplemental table 5. Prevalence of CT angiography–detected atherosclerosis and CAC in REPRIEVE vs SCAPIS vs PROMISE participants without established coronary heart disease who underwent successful coronary CT angiography and CAC scoring.

|  | **REPRIEVE** | | | | | **SCAPIS** | | | **PROMISE** | | | | |
| --- | --- | --- | --- | --- | --- | --- | --- | --- | --- | --- | --- | --- | --- |
|  | **40 to 44 y** | **45 to 49 y** | **50 to 54 y** | **55 to 59 y** | **60 to 64 y** | **50 to 54 y** | **55 to 59 y** | **60 to 64 y** | **45 to 49 y** | **50 to 54 y** | **55 to 59 y** | **60 to 64 y** |  |
| **CTA** | | | | | | | | | | | | | |
| Sample size | 110 | 212 | 237 | 139 | 49 | 8626 | 8317 | 8239 | 173 | 523 | 563 | 430 |  |
| Any form of atherosclerosis, n (%) | 33 (30.0) | 92 (43.4) | 121 (51.1) | 82 (59.0) | 34 (69.4) | 2578 | 3565 | 4459 | 91 (52.6) | 253 (48.4) | 318 (56.5) | 282 (65.6) |  |
| Any stenosis ≥50%, n (%) | 1/109 (0.9) | 7/208 (3.4) | 6/233 (2.6) | 8/136 (5.9) | 2/49 (4.1) | 237 | 426 | 654 | 16 (9.3) | 40 (7.7) | 66 (11.7) | 60 (14.0) |  |
| Any plaque non-calcified, n (%) | 25 (22.7) | 78 (36.8) | 103 (43.5) | 64 (46.0) | 28 (57.1) | 519 | 716 | 867 | 83 (48.0) | 225 (43.0) | 284 (50.4) | 240 (55.8) |  |
| CAC | | | | | | | | | | | | | |
| Sample size | 99 | 205 | 229 | 129 | 48 | 8563 | 8268 | 8183 | 149 | 469 | 480 | 377 |  |
| 0, n (%) | 79 (79.8) | 143 (69.8) | 150 (65.5) | 71 (55.0) | 20 (41.7) | 6175 (72.1) | 4882 (59) | 3900 (47.7) | 82 (55.0) | 264 (56.3) | 240 (50.0) | 146 (38.7) |  |
| >0, n (%) | 20 (20.2) | 62 (30.2) | 79 (34.5) | 58 (45.0) | 28 (58.3) | 2388 (27.9) | 3386 (41) | 4283 (52.3) | 67 (45.0) | 205 (43.7) | 240 (50.0) | 231 (61.3) |  |
| 1-400, n (%) | 20 (20.2) | 59 (28.8) | 75 (32.8) | 56 (43.4) | 26 (54.2) | 2280 (26.6) | 3151 (38.1) | 3741 (45.7) | 61 (40.9) | 182  (38.8) | 205  (42.7) | 176 (46.7) |  |
| >400, n (%) | 0 (0.0) | 3 (1.5) | 4 (1.8) | 2 (1.6) | 2 (4.2) | 108 (1.3) | 235 (2.8%) | 542 (6.6) | 6  (4.0) | 23 (4.9) | 35 (7.3) | 55 (14.6) |  |
| CAC, median (IQR) | 0.0 (0.0-0.0) | 0.0 (0.0-2.4) | 0.0 (0.0-8.8) | 0.0 (0.0-62.2) | 8.5 (0.0-85.9) | 22 (5-74) | 29 (8-106) | 52 (13-176) | 0  (0.0-38.0) | 0.0  (0.0-42.6) | 0.2  (0.0-78.1) | 15.0  (0.0-164.2) |  |
| CTA and CAC | | | | | | | | | | | | | |
| Sample size | 79 | 143 | 150 | 71 | 20 | 8563 | 8268 | 8183 | 82 | 264 | 240 | 146 |  |
| Any plaque non-calcified among patients with CAC=0, n (%) | 9 (11.4) | 27 (18.9) | 33 (22.0) | 17 (23.9) | 4 (20.0) | 222 (2.6) | 196 (2.4) | 180 (2.2) | 11 (13.4) | 23 (8.7) | 35 (14.6) | 15 (10.3) |  |

CAC=Coronary Artery Calcium; CTA=Computed Tomography Angiography.

**SUPPLEMENTAL FIGURES**

**Supplemental figure 1.A.** Presence of any coronary atherosclerotic plaque of REPRIEVE vs SCAPIS vs PROMISE participants without established coronary heart disease as stratified by age.


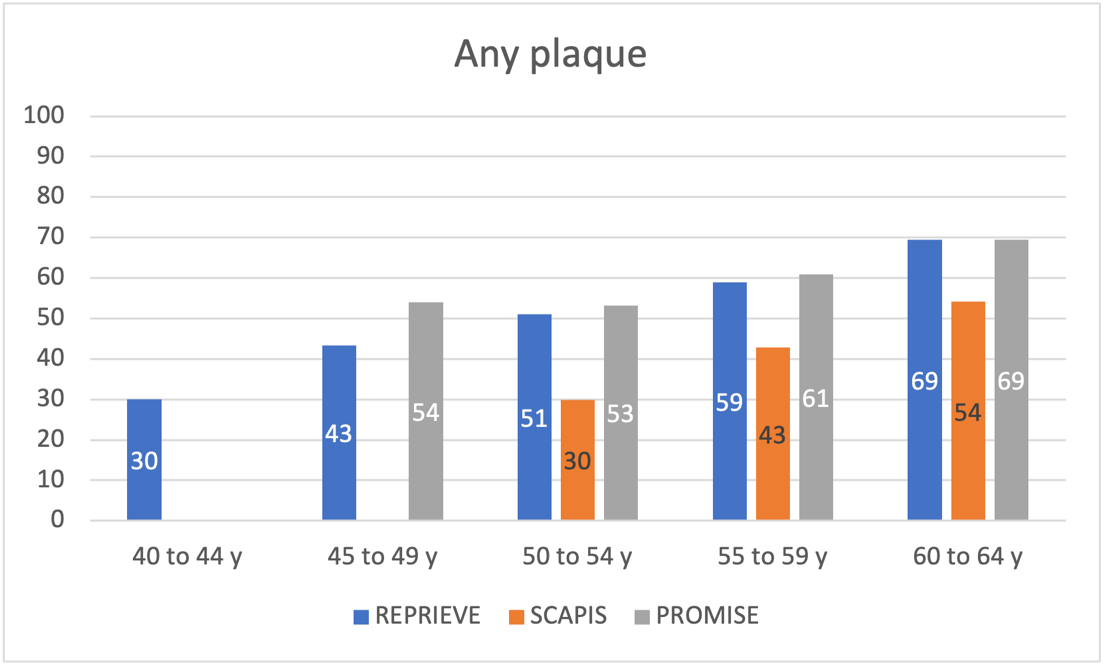


**Supplemental figure 1.B.** Presence of any coronary atherosclerotic plaque of REPRIEVE vs SCAPIS vs PROMISE participants without established coronary heart disease as stratified by age in men.

**
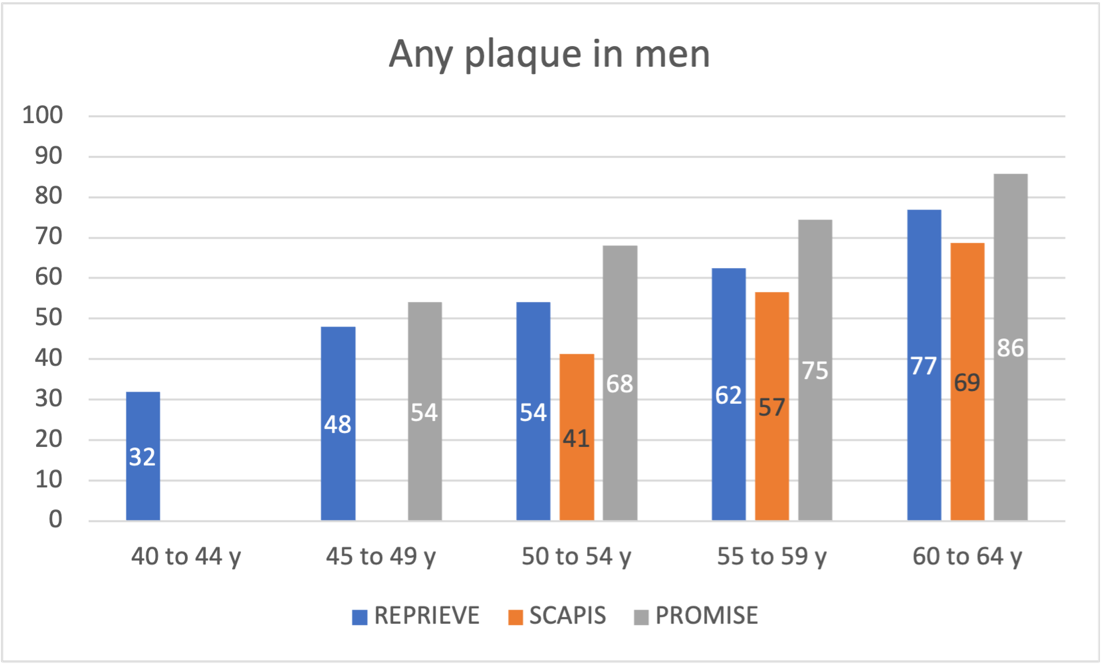
**

**Supplemental figure 1.C.** Presence of any coronary atherosclerotic plaque of REPRIEVE vs SCAPIS vs PROMISE participants without established coronary heart disease as stratified by age in women.

**
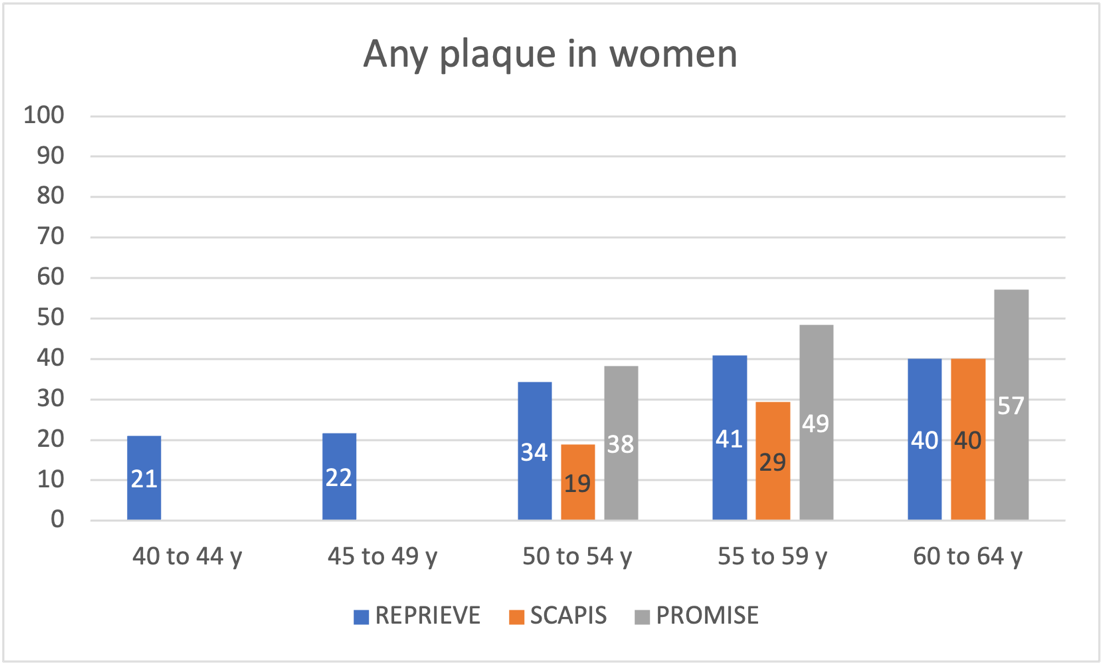
**

CAD: Coronary Artery Disease.

**Supplemental figure 2.** Presence of non-calcified atherosclerotic plaque of REPRIEVE vs SCAPIS vs PROMISE participants without established coronary heart disease stratified by age.


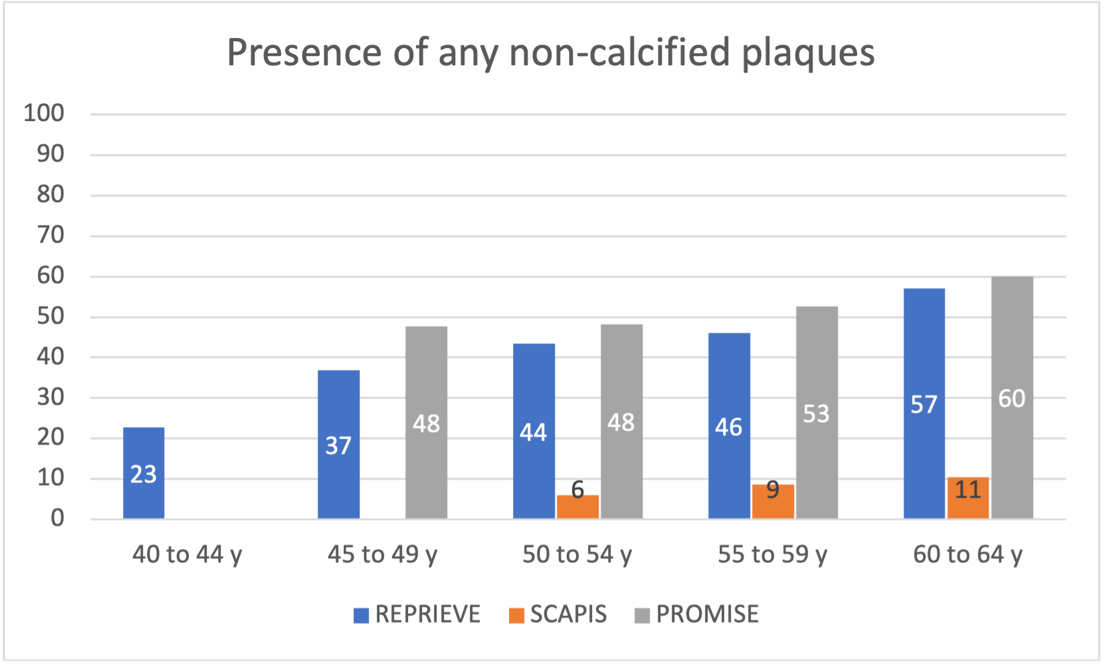


CAD: Coronary Artery Disease.

**Supplemental figure 3.** Presence of any coronary atherosclerotic plaque of REPRIEVE vs SCAPIS vs PROMISE participants without established coronary heart disease as stratified by 10-year risk for atherosclerotic cardiovascular disease.


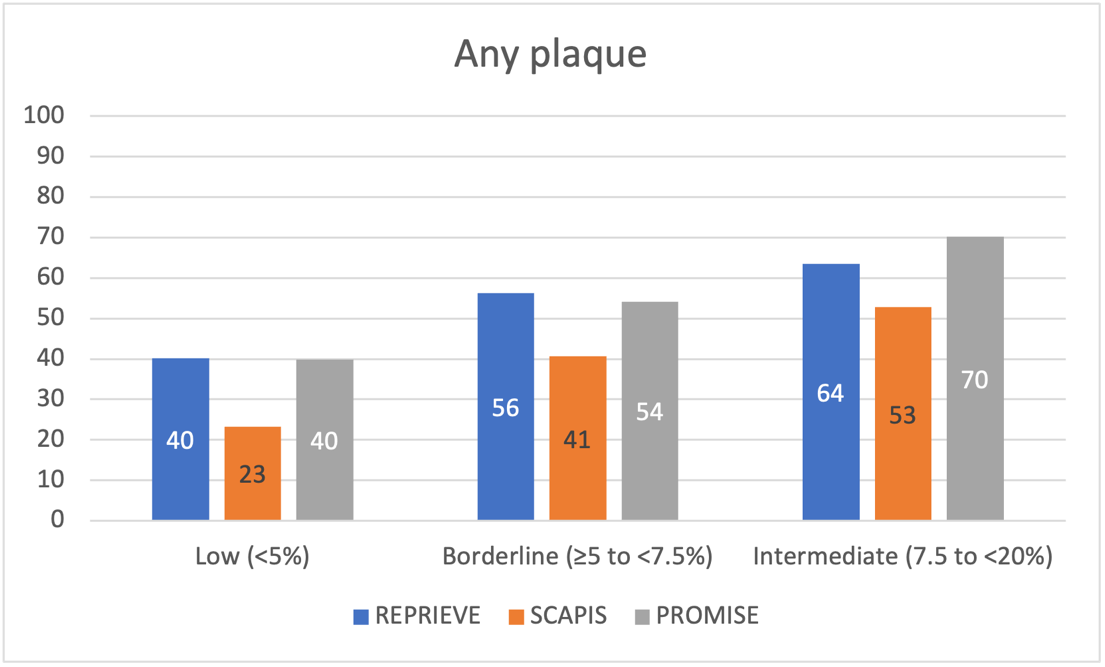


ASCVD: Atherosclerotic Cardiovascular Disease; CAD: Coronary Artery Disease.

**Supplemental figure 4.** Prevalence of any atherosclerotic plaque in the subgroup with CAC=0, stratified by 10-year risk for atherosclerotic cardiovascular disease.

**
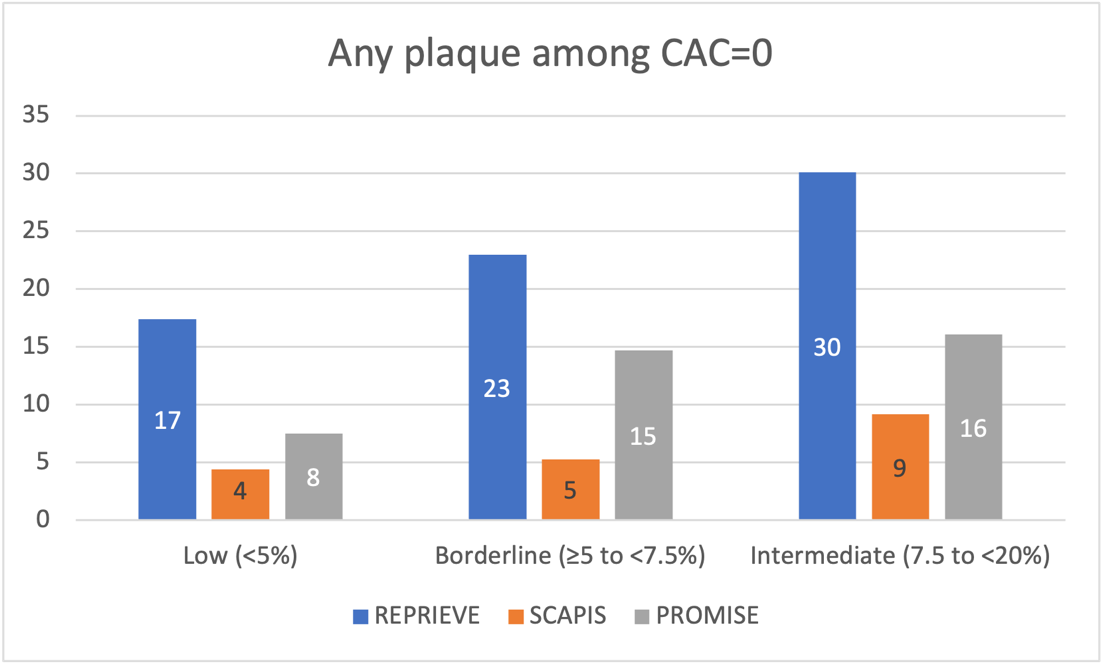
**

ASCVD: Atherosclerotic Cardiovascular Disease; CAC: Coronary Artery Calcium Score; CAD: Coronary Artery Disease.
